# On Some Points Connected with the Anatomy and Surgery of Inguinal and Femoral Herniæ

**Published:** 1833-10

**Authors:** 


*On some Points connected with the Anatomy and Surgery of Inguinal and Femoral Herniæ: being the Substance of the Lectures delivered in the Theatre of the Royal College of Surgeons, in February,* 1831. By G. T. GUTHRIE, F.R.S., Surgeon to the Westminster Hospital, &c. London, 1833. 4to. pp. 44.

THIS is a masterly though brief essay. Mr. Guthrie, in his introductory sentences, has enunciated in so lucid a manner the problem which it is his intention to demonstrate, that it would be an injustice to our readers to withhold the passage from them.

"I had the honour of delivering the Lectures in the Theatre of the Royal College of Surgeons, in February 1831, on the Anatomy and Surgery of Inguinal and Femoral Herniæ; and I took the liberty on that occasion to demonstrate the anatomy of some of the parts concerned in these derangements in a manner which had not been commonly adopted, but which I trust will be found more satisfactory than the method which is usually followed. A difference of opinion in regard to the structure of a part of the body is not of much real importance, unless it involves some practical point; and it is under this impression I venture to hope that the dissections made on that occasion, and the elucidations and explanations which resulted from them, have enabled me to remove a discrepancy that existed between the anatomy and surgery of these parts, which I had always pointed out in my private lectures, but was unable previously to explain.

"According to the prevailing opinion of modern surgeons, the parts through which an inguinal hernia passes or proceeds, have little or nothing to do with the causes of strangulation; which are supposed to depend upon certain circumstances connected with the state of the protruded parts themselves, rather than upon any positive contraction or diminution of the size of the aperture through which they pass. If it had been generally demonstrated or believed that the inner or superior opening of the inguinal canal was a muscular opening or split, through or between the fibres of which the protrusion took place, there would have been little difficulty or hesitation in attributing the cause of strangulation to a sudden or irregular contraction of these fibres: but as such fibres were not believed to exist, or were supposed only to exist on the upper part, the impossibility of any circular contraction was necessarily inferred; and other causes of strangulation were sought for in the ingenuity of those individuals whose attention was devoted to the subject. I am quite aware, that these individuals have been and are among the number of the most celebrated anatomists and surgeons in Europe; that any effort to add to their labours may be considered an act of supererogation; and that any attempt to differ in opinion from them will be thought, perhaps, a matter of vanity. Facts have, however, forced themselves upon me so strongly, that I could not help acknowledging their influence; and it will be for those who doubt, to investigate this subject by their aid, and, by a similar patient inquiry, to confirm or confute the opinions I have founded upon them; viz. that the inner or superior opening of the inguinal canal is a muscular opening, or rather split, capable of a great degree of contraction, which is usually the cause of strangulation in cases of recent herniæ, and is by no means an infrequent one even in older ones. Before I was enabled to demonstrate the muscular structure of these parts, I had had the opportunity of examining the bodies of two persons who had died from strangulated herniæ, in both of whom the stricture on the intestine had been so great, that a common silver probe could not be easily passed in the canal of the gut. The last case was that of a person who had been operated upon, and died shortly afterwards; the intestine had been returned into the cavity of the abdomen, and was found lying behind the inner ring, with a narrow but deep indentation around it, marking the place at which the stricture had existed, and through which a probe could only be passed by dilating the contraction. I showed the preparation at my lecture, and declared, what I believe to be true, that this could only have taken place from some direct muscular pressure from without, and not from any congestion or dilatation from within or below the stricture. I also acknowledged that I could not show, by dissection, in what manner this contraction had taken place, or by what parts it was effected. I have always, however, continued to impress upon the minds of the gentlemen attending my lectures for the last ten years, that the principal cause of strangulation in recent hernia was a contraction of the superior or internal opening of the inguinal canal, the cause and nature of which I could not satisfactorily explain; that it was, therefore, a point in anatomy deserving investigation, for a discrepancy of this kind could not exist in nature; and that there must be something defective in our knowledge of the subject." (P. 1.)

Mr. Guthrie then gives (from S. Cooper's Surgical Dictionary,) the conflicting opinions of the most eminent surgeons of Europe on the anatomy of the parts concerned in inguinal hernia, and says,

"I hardly dare venture to give the reason which in my mind has led to the great apparent discrepancy of opinion which exists between so many able men on so plain a matter of fact. It is possible that it may have arisen from the great minuteness with which it has been attempted to describe parts that scarcely deserve it, especially the fascia transversalis, and from the great variety which exists in the formation of several of the principal parts.

"Sir Astley Cooper first gave to the fascia the name of fascia transversalis, and drew attention to it in so marked a manner as to attract that of other anatomists. Jules Cloquet made it an object of particular study, and Blandin and Velpeau consider it to have been more accurately investigated by him, on which account I have transcribed his account of it, page 5. Cloquet says, that he had not seen Sir Astley Cooper's work, but had formed his ideas of it from that of Mr. Lawrence; and it appears to me that he, Blandin, and Velpeau, have fallen into a misapprehension on the subject, common to many of our own countrymen, who have described that to be fascia transversalis alone, which is in reality the tendon or aponeurosis of the transversalis muscle implanted upon it; for without such misapprehension it is impossible to say that the spermatic cord always lies on the fascia transversalis in any part of its course after it has passed the superior opening of the inguinal canal, and has emerged from under the fleshy fibres of the transversalis muscle; nevertheless this is said to be the case. The statement made by Cloquet, Blandin, and Velpeau, that the fascia transversalis arises from the inner edge of Poupart's ligament by a reflection upwards of that part, whilst Sir Astley Cooper describes it as passing beneath to form the sheath of the femoral vessels is another cause, I suspect, of the misunderstanding which has taken place. Cloquet, who wrote from very laborious observation, says, in a note at page 26 of his work, and in addition to the observations I have quoted, page 6, 'that very often the fascia transversalis is evidently formed of two aponeurotic layers, which are united on a level with the top of the crural arch. Of these the anterior comes from the arch itself (Poupart's ligament), the posterior being only a continuation of the fascia iliaca, which quits the iliac muscle to ascend upon the anterior wall of the abdomen. These two layers thus reunited proceed back to back between the transversalis muscle and the peritoneum. It is easy to separate them on the outside of the superior opening of the inguinal canal, but on the inside and around it they are intimately united. When this formation is met with, the posterior layer passes usually behind the rectus muscle in its way to the linea alba, whilst the anterior one is continuous with the edge of the tendon of the rectus. The epigastric artery is sometimes posterior, sometimes anterior, and sometimes even between these two layers.' This description, which Cloquet gives as of an accidental occurrence, is, in my opinion, that which most frequently takes place; and if the fascia transversalis be said to be composed of two layers, the anterior being fibrous, the posterior cellular, much confusion will be avoided.

"The division which is made of the fascia transversalis into two parts, where it lines the wall of the abdomen, one being called external, the other internal, or anterior and posterior by Sir Astley Cooper, (but which are not the anterior and posterior layers of Cloquet,) the spermatic cord passing between them through an opening, which is named the superior opening of the inguinal canal; is also a fertile source of inconvenience to the student, who will seek in vain for any such opening. If he is taught to consider the fascia transversalis as a sheet of condensed cellular membrane divisible in some parts into two layers, passing upwards from Poupart's ligament to fortify the peritoneum, he will readily understand it; and if he is shown that at a certain spot it becomes much thinner and allows the spermatic cord to pass through, he cannot fall into any misapprehension. This part is not however an opening; it is merely the thin portion of the fascia which, as the testis escaped from the abdomen, was carried forward by that gland, and is now seen attaching itself to the spermatic cord. If this cord be drawn down and an incision be made around it close to where it is attached to the peritoneum, a sort of ring is formed, and if the finger be introduced, the thin part can be stretched or torn, until the firm internal edge of the denser anterior layer of fascia transversalis can be distinctly seen, having the epigastric artery a little to its inner side. The outer side of the ring is not so well marked, and the hole thus made by the finger is usually so large, and its outer edge so weak, as to occasion little fear of any great constriction being made by it on any portion of the contents of the abdomen which may be protruded through it. It is therefore not the part which constitutes the stricture at what is called the inner ring." (P. 10.)

Although hernia is one of the commonest of all infirmities, so frequent indeed, that it has been conjectured that one sixth of the whole population of the kingdom is afflicted with it, and though, consequently, the opportunities of dissecting a hernial sac are innumerable, yet doubts still linger round this part of surgery, and throw their cold shade over the attempts to explain the exact nature of these morbid protrusions. Our author observes:

"From the misapprehension which has taken place with reference to the inferior portion of the transversalis muscle, the coverings which a hernia of direct descent receives at this part has been a matter of doubt. Mr. S. Cooper gives (page 660 of his Dictionary,) the opinions upon this point of Messrs. Hesselbach, A. Cooper, Cloquet, Lawrence, and Stanley, leaving it, however, undecided whether the covering or investment is or is not formed as, he says, Sir Astley Cooper is reported to have described it in his lectures, viz. one half by the tendon of the transversalis, and the other half by the fascia transversalis. According to my version of the anatomy, it appears to me quite clear that there are two investments, one formed of the two layers of the fascia transversalis, and another external to that formed by the tendon or aponeurosis of the transversalis. The statement said to be made by Sir Astley Cooper can only be correct when there is no inferior portion of muscular fibre or of aponeurosis to the transversalis muscle, and which is sometimes wanting, although rather as an exception to the general rule, than as the general rule itself, or when the insertion of the superior fibres of the transversalis is effected by a narrow tendon. In the hernia of direct descent, or the internal inguinal hernia of Hesselbach, the coverings, when enumerated from within, are, the peritoneum, fascia transversalis, tendon of the transversalis, and tendon of the internal oblique more or less conjoined, the intercolumnar fascia, the superficial fascia and integuments. As this hernia passes to the inside, and rather underneath the spermatic cord, it does not receive a covering from the cremaster. The external inguinal hernia, or of oblique descent, lies upon or above, and to the outside of the spermatic cord; the internal inguinal hernia, or of direct descent, lies to the inside of, or below and underneath the cord, constituting the principal features of diagnosis, especially in old herniæ. In the latter species, or of direct descent, the internal oblique muscle may not always be inserted low enough down towards the pubes, so as to give a covering to the hernia, which then only protrudes, or carries before it the transversalis. This fact will not however be discovered in operating; for the pressure on the parts causes such a consolidation of them, that the two tendinous expansions, when they exist, become so closely united as to form but one covering. When enumerated from without, the coverings are the integuments, superficial fascia, intercolumnar fascia, the tendinous expansions of the internal oblique and transversalis muscles when they exist, the fascia transversalis, and peritoneum.

"The internal oblique muscle would in the male form a layer of muscular and tendinous fibres external to the transversalis, and a complete support or covering to this part of the abdomen, if it were not for the opening to admit of the descent of the testis and the passage of the spermatic cord. The gubernaculum testis, a part of original formation, is supposed to possess the power of drawing down the testis through this opening, which I very much doubt. I believe that the testis descends or ascends, as the case may be, at the proper period, for the same reason that a child is usually born at nine months in preference to any other period of utero-gestation, which is, as Avicenna says, by the will of God. The office of the gubernaculum appears to be to keep a passage open which might otherwise be closed, if it were not occupied in this manner, rather than to operate on the testis by any contraction of its substance. As the testis passes through the transversalis muscle it may bring down with it any fibres which lie in its way; and when this occurs, the transversalis is found to be united at this part to the internal oblique, and the fibres thus brought down assist in forming the cremaster muscle, which is nothing more than a certain portion of the lower edge of the internal oblique caught by the testis and carried before it. The fibres caught on the centre of the testis are carried down with it into the scrotum by a gradual elongation, so that they form a sort of sling around the testis, which supports and can raise it towards the abdominal ring, whilst those fibres which are only entangled on its anterior and posterior surfaces, form arches, which descend before or behind according to the situation of the points of entanglement. The cremaster muscle is then a portion of the under edges of the transversalis and of the internal oblique muscles, arising also from Poupart's ligament by its under edge, until it is carried away by the descent of the testis; but the point of insertion of these fibres remains the same, viz. into Poupart's ligament near the pubes, after forming the sling and the arches above described, and which point of insertion was considered by Scarpa and others until the time of Cloquet, to be another or second origin, which is evidently an error. When the testis is detained in the abdomen, it is not for the want of an opening in the transversalis or internal oblique muscles, but for some reason which has not yet been sufficiently explained, as the person commonly suffers from a hernial protrusion, the consequence of the part being less defended than usual by the natural structures.

"The anatomy of the inguinal canal from without inwards is as follows. The tendon or aponeurosis of the external oblique muscle being turned down upon the thigh, the spermatic cord is seen lying in the inguinal canal, embraced by the cremaster muscle, which passes down upon it. The lower edge of the internal oblique separating from the cremaster which is a part of it, passes on to form its share of the sheath of the rectus. The cremaster being cut across and turned upwards, the spermatic cord is seen passing from under the fleshy edge or through the split of the transversalis muscle, and having beneath it another fleshy or aponeurotic portion of the same muscle descending to be inserted into Poupart's ligament. It is on this part that the cord lies, and it forms of course the posterior wall of the inguinal canal, having behind it more or less closely attached the fascia transversalis. If the internal layer of fibres of the transversalis curve downwards to be inserted into Poupart's ligament after passing over the cord, this part lies more decidedly upon them, and not on the fascia transversalis. See Plate III. fig. 1." (P. 16.)

In very old herniæ, the ring or opening is no longer affected by the contractions of the transversalis; and bleeding to syncope, which is so useful in the strangulation of a recent hernia, is prejudicial in an old one. In the latter case, "the first evil is from congestion and distention; the first stage is that of incarceration, followed ultimately by inflammation, first shown by pain at the umbilicus, and, after a greater or less lapse of time, by pain in the part, but which is never so acute as in the recent cases." (P. 22.) Mr. Guthrie has seen a case of this kind which had lasted three days, and terminated favourably.

"The swelling was large and very hard; but as the integuments were thin, and the part not painful, it admitted of accurate examination. The hardness appeared to be dependent on a collection of solid fæcal matter, and after some trials a portion of it at the upper part was broken, and pressed through the external ring. This was followed by a second, and so on in succession until the whole was pushed into the abdomen, with the intestine which had contained it. The symptoms immediately subsided, and a dose of purgative medicine completed the cure. The openings in the abdomen through which the parts protruded were in this case entirely passive." (P. 26.)

The regret we feel that our limits do not permit us to make any more extracts, is somewhat diminished by the probability that Mr. Guthrie's essay is already in the hands of half the operating surgeons in England. It is not only illustrated, but embellished with several handsome plates.

